# Noise Disturbance and Potential Hearing Loss Due to Exposure of Dental Equipment in Flemish Dentists

**DOI:** 10.3390/ijerph18115617

**Published:** 2021-05-24

**Authors:** Michael Dierickx, Suzanne Verschraegen, Els Wierinck, Guy Willems, Astrid van Wieringen

**Affiliations:** 1 Department of Oral Health Sciences-Dentistry, KU Leuven, University Hospitals Leuven, 3000 Leuven, Belgium; 2 Department of Oral Health Sciences-Orthodontics, KU Leuven, University Hospitals Leuven, 3000 Leuven, Belgium; verschraegensuzanne@hotmail.com (S.V.); guy.willems@uzleuven.be (G.W.); 3 Department of Oral Health Sciences-Dentistry, KU Leuven, 3000 Leuven, Belgium; els.wierinck@kuleuven.be; 4 Research Group Experimental Oto-rhino-laryngology, Department of Neurosciences, KU Leuven-University of Leuven, 3000 Leuven, Belgium; astrid.vanwieringen@kuleuven.be

**Keywords:** occupational noise, digit triplet test, pure tone audiometry, noise-induced hearing loss, dentistry

## Abstract

Long-term exposure to occupational noise is often associated with noise-induced hearing loss (NIHL) among dentists. This study aims to investigate potential hearing loss (HL) and self-reported annoyance as a result of exposure to noise produced by contemporary dental equipment. Methods: Three cohorts participated: 53 dentists with more than 5 years of service, 47 dentists with at most 5 years of service, and 53 pharmacists as controls, age and gender-matched to the first group. After the exclusion of one person, the hearing of 304 ears was screened with the Flemish version of the digit triplet in noise test (DTT). If screening failed, otoscopy and pure tone audiometry (PTA) were performed for both ears. Furthermore, general information, knowledge, exposure, annoyance, general health, and preventive measures were assessed with a custom-made questionnaire. Results: NIHL did not occur significantly more often with dentists than with controls. However, dentists revealed a significantly higher annoyance (related to the noise from their equipment) and reported more complaints than the pharmacists. All three groups indicated lack of knowledge on hearing care. Conclusions: While noise levels in contemporary dentistry are not harmful and do not induce NIHL, the sounds emitted by the devices are disturbing and affect mental health. This study calls for increased awareness of the consequences of sound exposure and stresses the need to monitor and protect the hearing of dentists regularly.

## 1. Introduction

Occupational NIHL accounts for 7% to 16% of disabling HL [1,2,3]. It is caused by chronic and excessive exposure to hazardous levels of noise and is characterized by permanently reduced sensitivity (>20 dB HL) in the 3–6 kHz range, with normal sensitivity at lower and higher frequencies [3].

It is commonly known that dental professionals are exposed to noise related to their work coming from various sources: aspirators, ultrasonic scalers, high- and low-speed handpieces, turbines, suction devices, etc. Noise is an unwanted, disturbing sound, either in frequency, level, duration, or a combination of these. In the past, the emitted levels often exceeded 80 dBA [4], especially of the high-speed handpieces, and dentists were at substantial risk of developing HL as a result of too much exposure to the noise of their equipment [5,6,7]. Although some studies report that the noise levels of dental equipment may still provoke HL [8,9,10,11,12,13,14,15], the exposure levels of contemporary dental equipment are generally within the limits set by the National Institute for Occupational Safety and Health (NIOSH) that recommends limiting noise exposure to 85 dBA at 40 h per week (e.g., [16,17]). This is possible due to technological improvements of the high-speed handpieces. As an example, sound levels of the new preclinical lab for dentistry at the university hospital Leuven (Belgium) are in the order of 72 dBA for 1 chair and 74 dBA for more chairs (internal document). These noise levels should not induce HL [18,19,20,21,22].

While the exposure levels of contemporary dental equipment may have become less hazardous in time, occupational HL remains a matter of debate. Some studies have reported poorer hearing thresholds for dental professionals compared to controls [11,13,14,23,24,25], while others report similar hearing thresholds [15,26,27,28]. Audiometric patterns generally reflect a mild hearing impairment between 2 and 6 kHz, but by no means with all dental professionals (e.g., [14]). Potential HL has also been attributed to specific dental equipment, such as the high-speed handpieces mentioned before [13]. However, across studies, data regarding dental equipment, often linked to the specialism, are not consistent. For instance, it has been reported that prosthodontists have the poorest hearing thresholds at the mean frequencies of 500–2000 Hz and 3000–6000 Hz when compared to dentists and dental nurses [24], while higher noise levels have also been reported for pediatric dentistry [16,18], even up to 112.9 dBA when children were crying during treatments [21]. Since prosthodontists are also general practitioners and therefore use handpieces and suction devices, most of the reported differences may not be due to the specialism itself but possibly due to a combination of other factors (including age and length of service).

Length of service is also mentioned as a factor for NIHL in dentists in several studies [11,29]. Ma et al. revealed that dental practitioners with more than 10 years of experience and more than 8 h of daily work have the highest risk of hearing impairment [30]. Similarly, Gonçalves showed that dentists with more than 10 years of work experience have a higher risk of developing hearing impairment between 500 and 1600 Hz than their control group [27]. While Khaimook also mentions that HL was significantly related to work tenure longer than 15 years, they did not observe differences between dental personnel and controls [31]. In general, the interpretation of the data is complicated by the fact that persons with several years of experience are presumably experiencing (emerging) age-related hearing loss (ARHL). ARHL (presbycusis) sets in around middle age, displaying a reduced sensitivity in the high-frequency range. This reduced sensitivity in the high-frequency region increases with age [32]. Finally, it is also important to bear in mind that observed NIHL does not have to be caused by exposure to the noise by the dental equipment, but it could be due to other non-occupational factors, such as exposure to other noise or ear diseases [33].

In addition to causing hearing impairment, noise can also affect human functioning, as is well documented for traffic and aircraft noise [34,35,36,37,38]. This may be especially true for the dental profession which, by itself, is stressful, with strict working schedules, dealing with anxious patients, and/or painful treatments. Common health problems among dentists include low back pain, impaired vision, allergies, stress, fatigue, headache, annoyance, and tinnitus [8,22,25,39,40].

Methodological differences between the above-mentioned studies complicate interpretation regarding potential harm and disturbance caused by exposure to occupational noise in dental clinics that comply with the standards set by the NIOSH. On top of this, most studies focus on either the hearing thresholds or self-reported complaints and fail to provide a comprehensive analysis of the causes of the observed NIHL, which may not be linked to the dental occupation.

The objective of this study is to investigate potential HL and self-reported annoyance as a result of exposure to noise produced by contemporary dental equipment. To control for comorbidities, objective hearing data and subjective reports were obtained from three different cohorts: dentists who had less than or equal to five years of professional experience (≤5 years service), dentists with more than five years of professional experience (>5 years service), and pharmacists, who were age and gender-matched to the second group of dentists. Hearing data were complemented with extensive survey data documenting exposure to noise, self-reported hearing and health issues, complaints, and knowledge of HL and hearing protection. We expect HL to occur more frequently in the older cohorts than in the younger ones, however, not necessarily due to exposure to occupational noise because of contemporary standards and technological advancements. We also expect that dentists will report higher disturbance to noise than pharmacists and that older persons might experience more difficulty suppressing noise than younger ones. Finally, we expect knowledge on hearing and HL to be limited in all three cohorts, since these factors are usually not taught at university.

## 2. Materials and Methods

### 2.1. Participants

Three different groups of active professionals participated in this study out of interest, without a specific demand of care: (1) dentists who had been practicing for more than five years (>5 years service, *n* = 53, average age 47 years), (2) dentists who had been practicing less than or equal to five years (≤5 years service, *n* = 47, average age 26 years) and (3) pharmacists as controls for the dentists with >5 years service (*n* = 53, average age 46 years). The 5-year cutoff was a pragmatic choice, based on the available study samples, i.e., postgraduate students (less than or equal to 5 years of training) and their internship supervisors who had to have more than 5 years of training. Pharmacists were chosen, as they are less exposed to occupational noises than dentists, while their medical knowledge and years of education are comparable to those of dentists. The latter two were necessary to fill out the survey. Pharmacists were age and gender-matched to the first group (see Table 1). The 153 participants were mainly recruited during postgraduate meetings. There were no exclusion criteria.

### 2.2. Hearing Screening

Hearing assessment followed a two-stage process: hearing screening and PTA. The hearing was screened with the Dutch/Flemish version of the digit triplet test, which is a paradigm with high sensitivity and specificity for detecting sensorineural HL [41]. Three speech digits are presented in noise, and the signal-to-noise ratio (SNR) was varied adaptively until a speech reception threshold (SRT) was achieved. This is the SNR at which 50% of the triplets are identified correctly. The DTT was carried out for both ears in all participants using a tablet (Lenovo) with sound transmitted to a calibrated Peltor H7A headphone. Participants were seated in a quiet office-like room. After familiarization with five times three digits, the SRT per ear was determined by presenting a set of 17 triplets with a broadband masking noise using a one-up-down adaptive procedure of which the first triplet was presented at −2 dB SNR. All digits per triplet needed to be identified correctly to decrease the SNR. The cutoff was set at −10.0 dB SNR [42], and persons who passed the test for both ears did not need to undergo any further hearing testing. The DTT is developed for hearing screening and is meant as a first triage. Per ear, it takes about 3 min to determine the SRT in steady-state noise. The DTT is less time-consuming than pure-tone audiometry and does not require a sound booth nor an experienced audiologist [41,42].

### 2.3. Otoscopy and PTA

Those participants who failed the DTT for at least 1 ear had to undergo an otoscopy to check for ear blocks, perforation or fibrosis of the tympanic membrane, and signs of otitis media. Afterwards, pure-tone air conduction thresholds were determined at octave frequencies ranging from 250 Hz to 8 kHz, including 3 kHz in a soundproof booth at the hearing lab of Experimental ORL. PTA was done bilaterally even if only one ear had failed hearing screening. This was done with an audiometer (Orbiter 922) according to the Hughson–Westlake five-up-ten-down method. For NIHL, the audiometric pattern had to adhere to the following 3 requirements: (1) a decrease in threshold of at least 20 dB HL at 3000, 4000, and/or 6000 Hz, (2) an HL at these frequencies greater than the age-related norm value, and (3) at least 10 dB HL poorer than the threshold at 8000 Hz [3]. As a result, the audiogram shows a notch, and both the shape and the depth (HL) of the notch are used to differentiate from, for example, presbycusis, which presents itself by a sloping decline from 4 kHz onwards.

### 2.4. Questionnaire

The custom-made questionnaire for both the dentists and the pharmacists was divided into two sections (Figure S1). The first section requested the participant’s name, workplace, and telephone number. This section was linked to a code written on the questionnaire and saved on a file solely available for the researchers. The second section of the questionnaire was divided into six parts. As we could not find a validated questionnaire on this topic, questions were derived from different questionnaires in the literature and piloted with colleagues of dentistry at UZ Leuven. The first part requested general information: gender, age, university and year of graduation, specialization, periods of inactivity as a dentist (or pharmacist), working alone or in a team, handedness (dentists only), working with visual magnification and brands of rotary instruments (dentists only). The second part examined general health: diagnosis of HL, ear operations, ear (related) disorders (family history, herpes zoster oticus, Ménière, otitis media, cholesteatoma, tumors in or around the ears, cleft lip, cardiovascular or neurological problems, diabetes), colds, and smoking. The third part documented the professional’s exposure to loud equipment (in the number of days per week, hours per day, and duration of intervals) as well as age and maintenance of rotary equipment (dentists only), exposure to air conditioning or music during their profession, exposure to non-occupational loud noises or ototoxic agents. The fourth part inquired about the annoyance of the participants: annoyance to noises in their workplace on a five-point Likert scale, whether noises become more annoying in time or whether they believe have influenced their hearing, whether their clients were anxious about these noises (dentists only) and whether they had complaints of which they thought these noises could be responsible for. The fifth part investigated the participant’s knowledge regarding HL in general and their interest in receiving more information about this subject through several media. The sixth and last part verified the use of preventive measures against loud noises: hearing protection devices, sound-absorbing materials, separate room for extractor systems (dentists only), and regular testing of the hearing. Approximately ten persons with missing data were contacted by mail or by telephone to complete their data.

### 2.5. Statistics

Statistics were performed using IBM SPSS 27 (IBM Corp., Armonk, NY, USA) [43]. One-way Analysis of Variance (ANOVA) was applied, with Bonferroni correction for multiple comparisons. Other comparisons required chi-square statistics. The level of significance was set at α = 0.05.

## 3. Results

### 3.1. Hearing Screening, Otoscopy and PTA

Of the group of dentists with >5 years service, two ears (one person) were excluded from further testing due to bilateral hearing aids (Table 1). In total, 33/104 ears of dentists with >5 years service, 40/106 ears of pharmacists, and 13/94 ears of dentists with ≤5 years service failed the hearing screening unilaterally or bilaterally. After otoscopy and PTA, 21 ears appeared to have normal hearing, i.e., hearing thresholds below or at 20 dB HL between 250 and 8000 Hz (eight ears of dentists with >5 years service, six ears of pharmacists, and seven ears of dentists with ≤5 years service). Since PTA was done bilaterally, 19 ears that had initially passed hearing screening presented an HL in one or more frequencies (nine ears, nine ears, and one ear for dentists with >5 years service, pharmacists, and dentists with ≤5 years service, respectively). Subsequently, a decline in hearing at one or more frequencies was established in 34/104 ears of dentists with >5 years service, 43/106 ears of pharmacists, and 7/94 ears of dentists with ≤5 years service.

### 3.2. Pattern of HL

**Figure 1:**
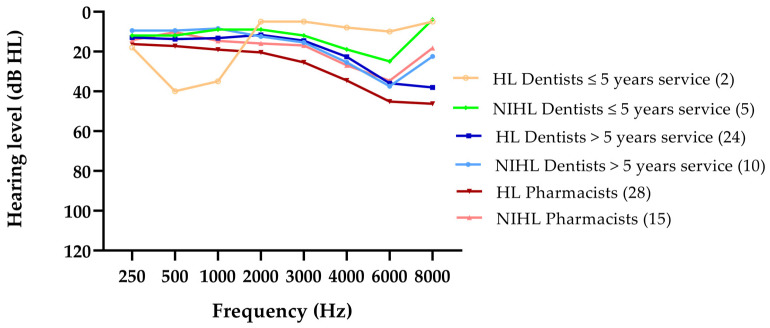
The two main patterns of audiometric hearing thresholds, NIHL and HL, for the two groups of dentists and the controls. For NIHL, the audiometric pattern had to adhere to the following 3 requirements: (1) a decrease in threshold of at least 20 dB HL at 3000, 4000, and/or 6000 Hz, (2) an HL at these frequencies greater than the age-related norm value, and (3) at least 10 dB HL poorer than the threshold at 8000 Hz. HL was defined as hearing thresholds above 20 dB HL at one or more frequencies between 250 and 8000 Hz. The number of ears is mentioned between brackets.

Subsequently, the audiometric patterns of the remaining 84 ears were analyzed in more detail to examine whether NIHL occurred more often with dentists than with pharmacists. Figure 1 displays two main patterns of audiometric thresholds per group: NIHL and HL. A pattern of NIHL is established for 10 ears in the group of dentists with >5 years service, 15 ears of pharmacists, and 5 ears of dentists with ≤5 years service (see numbers between brackets in the legend). The dip between 4 and 6 kHz decreased to, on average, 38, 35, and 25 dB HL for dentists with >5 years service, pharmacists, and dentists with ≤5 years service, respectively. Other HL was observed for 24 ears, 28 ears, and 2 ears, respectively, for the dentists with >5 years service, the (age-matched) pharmacists, and the dentists with ≤5 years service.

### 3.3. NIHL Due to Dentistry or Not?

The above-mentioned analysis indicates that relatively mild HL occurs in 84/304 ears of which 30/84 present a pattern of NIHL. In our sample, NIHL occurs somewhat more often for controls (pharmacists) than for dentists. NIHL is not only caused by exposure to occupational noise. Based on the questionnaire data (Table S1), participants from the three NIHL groups were further discarded if they had had ear operations; ear-(related) disorders (herpes zoster oticus, Ménière, otitis media, cholesteatoma, tumors in or around the ears, cleft lip, cardiovascular or neurological problems, diabetes); exposure to ototoxic agents or non-occupational loud noises (car racing, motorcycling, loud music, airplanes, speedboats, firing arms, gardening equipment, fireworks, explosions, etc.); abnormal otoscopy (cerumen, perforation, or fibrosis of the tympanic membrane, otitis media); see File S1, Table 1, and Table S1.

The remaining 5/104 ears of dentists with >5 years service and 2/94 ears of dentists with ≤5 years service remain with a clear NIHL pattern that cannot be explained by other factors than their profession (Table 1). Since this incidence is similar to that of the pharmacists/controls, i.e., 7/106 ears with a pattern of NIHL that cannot be explained by the factors presented in the questionnaire either, it remains unclear whether the NIHL patterns observed with the dentists are caused by the noise emitted from the dental equipment. Consequently, the profession of dentist does not seem to induce a higher incidence of NIHL than pharmacists.

### 3.4. Survey

#### 3.4.1. General Information

The survey data revealed that both the dentists with >5 years service and the pharmacists had been practicing their profession for a similar number of years, i.e., on average, 23.7 years (SD 10.8) and 22.2 years (SD 10.9), respectively. Furthermore, 13.5% of the dentists with >5 years service and 8.5% of dentists with ≤5 years service was left-handed. In addition, 67.3% of dentists with >5 years service and 51.1% of dentists with ≤5 years service used visual magnification (Table S1).

#### 3.4.2. Exposure to Noise

The survey showed that the average number of working days was similar for the three cohorts (approximately 4.7 days/week). The majority of dentists works 7–8 h a day. About 27% of dentists with >5 years service are exposed to occupational noise for more than 8 h a day compared to 13% of their younger colleagues. Most dentists (approximately 50%, both groups) are exposed to periods of 15–30 min of noise during their intervention; about 20% are exposed to 30–45 min of noise.

In both dentistry groups, most of the rotary instruments were between 1 and 5 years old and were maintained daily (Table S1). In total, 47 out of 52 dentists with >5 years service used at least one of the three most common brands in Flanders, i.e., W&H (W&H Dentalwerk Bürmoos GmbH, Bürmoos, Austria), Kavo (KaVo Dental GmbH, Biberach/Riss, Germany) and Bien-Air (Bien-Air Dental SA, Bienne, Switzerland), sometimes together with other brands (which are known to emit levels within the ranges set by NIOSH). Nearly 40% of dentists with ≤5 years service did not know which brands they used, but since they were working in the same practices as the dentists with >5 years service, we assume these brands were comparable.

On average, 87% of the dentists (both groups) had air conditioning in their workspace, and 77% (both groups) listened to music during practice. Few dentists and controls were smokers or past smokers. Each of the three cohorts reported substantial exposure to loud noises other than dental equipment, i.e., 33% divided over motorsports, loud music, firearms, gardening tools, fireworks, etc. (Table S1).

#### 3.4.3. Annoyance

When asked whether they were annoyed by the sound in their practice, the dentists with >5 years service scored, on average, 2.8 (SD 0.91), the pharmacists 2.1 (SD 0.84), and the dentists with ≤5 years service 2.9 (SD 0.9) on a Likert scale from 1 (never) to 5 (always). Even though the average values do not indicate extreme annoyance, a univariate ANOVA showed a significant effect of a group (F (2152) = 13.7, *p* < 0.001). Post hoc comparisons using Bonferroni correction revealed that the scores of the dentists and the pharmacists differed; however, they did not differ amongst the two groups of dentists.

At least 40% of dentists with >5 years service reported that noise became more annoying in time, which was a much larger percentage than that of pharmacists (8%) and dentists with ≤5 years service (13%). Approximately one-third of the older and more experienced dentists (33%) also believed that their profession altered their hearing compared to 9% of the younger dentists and none of the pharmacists. Moreover, nearly 60% of the dentists with >5 years service and 38% of dentists with ≤5 years service reported that they had patients who were anxious about the noises produced by professional equipment. The above-mentioned shows that occupational noise has an impact on the well-being and self-perception of the dentist.

#### 3.4.4. Complaints

**Figure 2:**
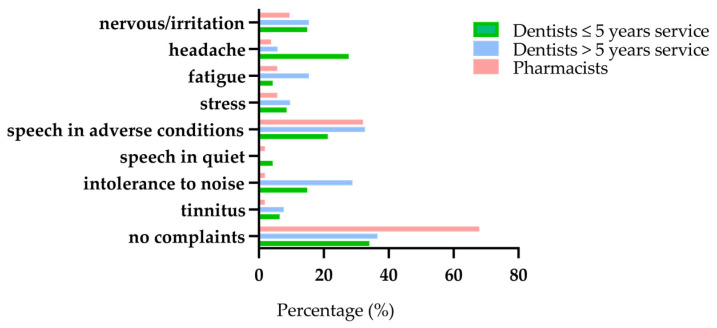
Potential complaints related to occupational noise exposure of the two groups of dentists and the controls.

The survey also tapped into potential complaints related to occupational noise exposure. Detailed responses are listed in Table S1, and the most common complaints are illustrated in Figure 2. A significantly higher percentage of pharmacists responded that they did not experience any complaints (68%) compared to the dentists with >5 years service (37%) and the dentists with ≤5 years service (34%). This was corroborated by a chi-squared test (χ^2^ = 9.8, df = 2, *p* = 0.007). Regarding the other questions, multiple responses were possible for those persons who experienced complaints. While relatively few dentists reported tinnitus (average 7% for both groups compared to 2% for the pharmacists), both dentists with >5 years service and dentists with ≤5 years service experienced significantly more intolerance to noise (average 22%) than pharmacists (2%, χ^2^ = 16.3, df = 2, *p* < 0.000).

Almost none of the persons in the three groups experienced difficulties understanding speech in quiet (<5%), while 32% of participants in both older cohorts experience difficulties understanding speech in adverse conditions (Figure 2), versus 21% for the younger dentists. Understanding speech in noisy conditions is not only associated with noise exposure but also with aging. This explains why the data are similar for the older dentists and pharmacists (χ^2^ = 1.8, df = 2, *p* = 406).

Although complaints related to stress and fatigue are limited, dentists with >5 years service experience significantly more fatigue than their peer pharmacists and the dentists with ≤5 years service (χ^2^ = 6.14, df = 2, *p* = 0.46). The younger dentists report significantly more headache (28%) than the other two groups (χ^2^ = 12.3, df = 2, *p* = 0.002), and both groups of dentists complain significantly more about nervousness/irritation (average 15%) than pharmacists (9%, χ^2^ = 8.3, df = 2, *p* = 0.014). These data suggest that dentistry is a demanding profession that weighs on the psychological functioning of young and middle-aged persons.

#### 3.4.5. Knowledge on HL and Hearing Protection

When asked about their knowledge on HL on a Likert scale from 0 (no knowledge of HL) to 5 (know everything of HL), the different groups reported a similar lack of knowledge. The average Likert score was 1.9 (SD 0.8) for dentists with >5 years service, 2.1 (SD 0.9) for dentists with ≤5 years service, and 1.8 (SD 0.9) for pharmacists (Univariate ANOVA, n.s.). Only 3% of the dentists (both groups) tested their hearing regularly, and 15% of them wore hearing protection devices for either private or professional reasons, compared to 13% and 25% of the control group. This higher percentage of the latter could be because pharmacists meet with sales representatives and sell products for hearing protection themselves. Finally, 96% of the dentists with >5 years service, 98% of the dentists with ≤5 years service, and 91% of the pharmacists indicated that they would appreciate more information about HL (Table S1).

Regarding preventive actions, 21% of the dentists with >5 years service, 11% of the dentists with ≤5 years service, and 4% of the controls reported the presence of sound-absorbing material in their workspace and almost 73% of the dentists (both groups) had placed their extractor systems in a separate room.

## 4. Discussions

The main aim of the study was to gain a thorough understanding of potential hearing impairment and health issues as a result of occupational noise in contemporary dentistry. In total, 304 ears were tested, which were divided over three cohorts: young and older dentists and a control group of pharmacists who are believed not to be exposed to occupational noise. Both the pharmacists and the older dentists had approximately the same age and had worked, on average, a similar number of years. All volunteers were active, healthy professionals who participated out of interest without a specific demand for care. Even in this population, the audiometric data already reveal mild to moderate HL in the three cohorts, and the self-report data present considerable annoyance and other mental issues with the dentists.

### 4.1. Hearing Loss

Of the 304 ears tested, 28% (84 ears) present a pattern of HL. Audiometric analysis showed that 30 ears have a pattern that resembles NIHL and that 52 ears showed other patterns of HL, mostly ARHL. One (younger) dentist has low-frequency HL. For NIHL, the threshold at 8000 Hz is better than that of lower frequencies in the high-frequency range. ARHL also produces a high-frequency HL but in a down-sloping pattern without better hearing thresholds at 8000 Hz. In general, HL is mild to moderate, although it is larger than expected for middle-aged persons [32]. Irrespective of the underlying cause, both NIHL and ARHL are sensorineural. While intense levels can cause immediate trauma to the cochlear hair cells and tissues in the inner ear, lower levels of noise result in the loss of synaptic connections between the inner hair cells and spiral ganglion neurons, causing cochlear synaptopathy [44,45]. Animal studies demonstrate that noise exposure can permanently damage the synapses between inner hair cells and auditory nerve fibers, even in the presence of intact outer hair cells and clinically normal audiometric thresholds. Since synaptopathy disrupts the afferent connection between the cochlea and the central auditory system, it is nowadays seen as a major cause of (emerging) speech understanding difficulties, and potentially of tinnitus and/or hyperacusis.

Mild HL is sufficient to prove a nuisance in certain communicative situations whether caused by noise or normal aging of the auditory system. From middle-age onwards, it is difficult to disentangle NIHL from ARHL, and subsequently, to decide whether a pattern of NIHL could have been caused by occupational noise or rather by other factors, also because it is known that noise is a modifiable risk factor of presbycusis.

Based on an evaluation of both the audiometric patterns and the responses on the self-report data, only five ears of older dentists, two ears of the younger cohort, and seven ears of pharmacists present patterns of NIHL that cannot be explained by external factors. In our study, the profession of dentist does not seem to induce a higher incidence of NIHL than controls. Our data are in line with previous reports [15,26,27,28,31], while others reported contrary results [11,13,14,23,24,25,46,47]. Since both older cohorts had been working for more than 20 years on average and show similar incidence of HL, our study cannot confirm that duration of exposure significantly affects hearing in dentistry, as was reported by Gurbuz, who found a significant correlation between the total working duration (years × days × hours) and the level of HL at all frequencies [11]. Rather, hearing worsens as a result of aging with diminishing hearing thresholds emerging at middle age [32]. Other discrepancies in findings between our research and studies can be explained by several factors, such as the use of older and more noisy equipment and/or higher environmental noise exposure [46,48,49]. In addition, the sensitivity to noise exposure is individually determined [50].

Our self-reported data also indicate speech understanding difficulties, especially in adverse listening conditions. This is presumably because of significant loss in the high-frequency region. A recent PNAS study employing both high-frequency audiometry and digits in noise (with low-pass filtering of the noise to detect high-frequency HL) showed that sound energy above 8 kHz contributes to speech perception in noise and that extended high-frequency loss predicts self-reported difficulty hearing speech [51]. The importance of measuring high-frequency thresholds for the early diagnosis of NIHL has been raised before [24,52].

### 4.2. Annoyance

Although dentists may not be at a larger risk of developing NIHL than other professionals, they are exposed to occupational noise and are thus also susceptible to other health problems. Our study revealed that both young and older dentists were significantly more annoyed than pharmacists about the noise in their workplace. These data are in line with those of earlier reports [22,25]. Annoyance is not only caused by the levels of exposure but also by other characteristics of the noise. In their health risk model, Ma et al. showed that the sharpness of the sound is a risk factor for headache, nausea, fatigue, hypertension, irritation, and tinnitus in the dental profession [30]. Therefore, not only the level of the noise but also its frequency spectrum should be taken into account when considering annoyance.

Our data confirm previous studies that the ability to suppress noise diminishes with age and that hearing impairment may have an aggravating effect on noise tolerance [53,54]. Interestingly, a study on self-reported annoyance with nearly 63,000 participants showed that middle-aged persons reported more annoyance than younger or older persons. This pattern was independent of noise exposure level and self-reported noise sensitivity [55]. In our study, 40% of middle-aged dentists indicated that the annoyance to occupational noises became more annoying in time, and 33% had the feeling that these noises changed their hearing (compared to 8% and 0% for the pharmacists, see Table S1). In addition, dentists report more complaints which they believed were a result of their profession (63% of dentists with >5 years service and 66% of dentists with ≤5 years service) than controls (32%). These mental health issues should not be neglected. Dentists should be made aware of them and take preventive measures, such as regularly monitoring their hearing, placing their extractor systems in a separate room, and wearing hearing protection devices. As foam or rubber earplugs may hamper communication, custom-filtered earplugs are advised. These allow accurate hearing at lower sound levels.

### 4.3. Exposure and Equipment

The NIOSH recommends limiting noise exposure to 85 dBA at 40 h per week. Especially, the high-speed handpiece and the aspirator may be hazardous to dentists. Most dentists in our study used rotary instruments from the brands W&H, Kavo, and Bien Air. The sound levels emitted from this equipment are well below the limits defined by the NIOSH [12,18,56,57]. Furthermore, used and worn rotary instruments could lead to higher noise levels due to bearing failure [12,57,58]. In our study, 81% of the older dentists stated that their rotary instruments were less than 5 years old and that their rotary instruments were maintained daily. Therefore, it is unlikely that these factors contribute to higher noise exposure in our contemporary dental practice. In addition, dentists are exposed to the noise for relatively short periods, ranging from 5 to 45 s [58].

In our study, 92% of the more experienced dentists reported having their compressor placed in a separate room. This implies that noise exposure due to compressors is limited, as was also confirmed by Gurbuz in 2013 [11]. Similarly, the fact that most of the dentists work in a team nowadays (90% and 98% for dentists with >5 years service and ≤5 years service, respectively) can only contribute to a slight increase of noise exposure [58].

As the result of the very low number of NIHL in our study, a breakdown in specialism in dentistry is not meaningful. Lopes et al. compared high-frequency hearing thresholds of dentists, dental nurses, and prosthodontists, and they reported that prosthodontists had poorer hearing than dentists at the mean frequencies of 500–2000 Hz and 3000–6000 Hz [24]. However, note that it is difficult to disentangle exposure to specialism, since prosthodontists mostly start as general practitioners. In our study, 56% of the dentists with >5 years service were prosthodontists, and 83% of the dentists with ≤5 years service were (still) general practitioners.

### 4.4. Hearing Care

While dentists may not be at immediate risk of developing hearing impairment, the daily exposure to noise warrants awareness of the long-term consequences of noise (both sensory and mental). Both dentists and other professionals indicate a lack of knowledge and mention that they are interested to learn more about this. For a few years, a class on hearing, NIHL, and prevention has been incorporated into the dentistry curriculum at the University of Leuven. In addition to raising more awareness about the consequences of hearing, it is highly recommended that dentists have periodic audiological evaluations and adhere to personal protection. Occupational and recreational NIHL can be avoided given sufficient knowledge on the topic.

Screening programs aim at awareness and early identification of individuals with HL. The DTT paradigm does not replace PTA but serves as an important triage. It has been translated and validated for many languages following the first Dutch version [59]. High sensitivity and specificity values of >80% are obtained; for a recent review, see Van den Borre et al., 2021 [41]. DTTs require only a minimum of linguistic and cognitive abilities, have a high measurement precision (test–retest reliability <1 dB), and can generally be conducted in less than five minutes [42]. In Flanders, the DTT is also used for hearing screening of school-aged children [60], but it is not yet implemented across the life span. In March 2021, the WHO launched the World Report on Hearing [61], presenting a global call for timely action to prevent and address HL across the life course. This includes a healthy lifestyle and easy accessibility to low-cost hearing screening at fixed intervals across the life course, empowering persons to act if needed [62].

## 5. Conclusions

In our study, dentists are not more subject to NIHL than pharmacists. Hearing impairment is relatively mild and not necessarily caused by the occupation. However, our study does add to the evidence that dentists risk health problems related to occupational noise exposure. Our study revealed a significantly higher annoyance and more self-reported complaints for the dentists than for the pharmacists. Moreover, they have limited knowledge of hearing care. Together, this calls for more awareness on the cascading consequences of NIHL and the implementation of regular hearing screening and preventive actions.

## Figures and Tables

**Table 1:** Main demographics of the two groups of dentists and the pharmacists.

Subject Characteristics	Dentists >5 Years Service (Ears = 106)	Pharmacists (Ears = 106)	Dentists ≤5 Years Service (Ears = 94)
Mean age in years (SD)	46.6 (10.5)	46.3 (10.8)	25.6 (3.1)
Range in years	29–66	29–64	22–36
Female (%)/male (%)	27 (51%)/26 (49%)	27 (51%)/26 (49%)	28 (60%)/19 (40%)
Excl. due to hear. aid (ears)	2	0	0
DTT passed (ears)	71	66	81
DTT failed (ears)	33	40	13
No HL after PTA	8	6	7
Occupational NIHL?	5	7	2
NIHL due to other causes	5	8	3
HL other than NIHL	24	28	2

## Data Availability

The data presented in this study are available upon request from the corresponding author. The data are not publicly available due to privacy reasons.

## Supplementary Materials

The following are available online at https://www.mdpi.com/article/10.3390/ijerph18115617/s1, Table S1: Summary of the characteristics of the questionnaire, File S1: Custom-made questionnaire for the dentists.
