# Supplementary material for: Noise Disturbance and Potential Hearing Loss Due to Exposure of Dental Equipment in Flemish Dentists
Source: Int J Environ Res Public Health. 2021 May 24;18(11):5617. doi: 10.3390/ijerph18115617 (PMC8197294; doi:10.3390/ijerph18115617)
Supplement: Supplementary file 1 [file ijerph-18-05617-s001.zip › ijerph-1187535-supplementary.pdf]

International Journal of
Environmental Research and Public Health

MDPI

Supplementary Table S1. Summary of the characteristics of the questionnaire

Subject characteristics	Dentists >5 years service (*n* = 53)	Pharmacists (*n* = 53)	Dentists ≤5 years service (*n* = 47)
**Part 1. General information**			
Gender			
Male	49.06%	49,06%	40,43%
Female	50.94%	50.94%	59.57%
Mean age ± SD	46.60 ± 10.46	46.28 ± 10.75	25.64 ± 3.13
Graduated at *			
Leuven	79.25%	73.58%	95.74%
Gent	5.66%	11.32%	0%
Brussels	13.21%	11.32%	0%
Others	3.78%	7.55%	4.26%
Years practising (mean ± SD)	23.67 ± 10.77	22.23 ± 10.89	1.04 ± 0.20
Specialization *			
Prosthodontics/esthetic	55.77%	NA	10.64%
Periodontology/implantology	5.77%	NA	0%
Endodontology	9.62%	NA	4.26%
Pedodontology	7.69%	NA	2.13%
General practitioner	21.16%	NA	82.98%
Others	1.92%	NA	4.26%
Working			
Alone	9.62%	9.43%	2.13%
In team	90.38%	90.57%	97.87%
Lefthanded	13.46%	16.98%	8.51%
Visual magnification	67.31%	NA	51.06%
**Part 2. General health**			
Diagnosis of hearing loss	11.54%	13.21%	2.13%
Past ear probl./ operations	15.38%	15.09%	21.28%
Ear disorders *			
Familial history	17.31%	18.87%	4.26%
Herpes z. w. postherp. neur.	0%	0%	0%
Ménière's disease	1.92%	0%	0%
Otitis media	1.92%	9.43%	4.26%
Cholesteatoma	0%	0%	0%
Tumors in or around ear	0%	0%	0%
Cleft	0%	0%	0%
Cardiovascular problems	3.85%	7.55%	0%
Neurological problems	0%	3.77%	0%
Diabetes	0%	0%	2.13%
Others	1.92%	1.89%	0%
No disorders	78.85%	67.92%	89.36%
Cold during testing	17.31%	5.66%	17.02%
Smoking			
Currently smoking	3.85%	1.89%	10.64%
Past smoker	11.54%	9.43%	2.13%
**Part 3. Exposure**			
Average days working	4.54	4.81	4.70
Exposure (h/day)			
Not exposed	0%	79.25%	0%
<1	0%	18.87%	0%
2-January	1.92%	0%	0%
3-February	1.92%	0%	0%
4-March	1.92%	1.89%	0%
5-April	5.77%	0%	19.15%
6-May	9.62%	0%	10.64%
7-June	19.23%	0%	17.02%
8-July	32.69%	0%	40.43%
>8	26.92%	0%	12.77%
Interval exposure (min)			

Not exposed	0%	79.25%	0%
<15	21.15%	16.98%	14.89%
15–30	50%	1.89%	59.57%
30–45	21.15%	0%	19.15%
45–60	1.92%	1.89%	6.38%
>60	5.77%	0%	0%
Age of RI (y)			
<1	5.77%	NA	10.54%
5-January	75%	NA	40.43%
>6	15.38%	NA	10.64%
Unknown	3.85%	NA	38.3%
Maintenance of RI			
Daily	80.77%	NA	65.96%
Few times/week	15.38%	NA	21.28%
Weekly	3.85%	NA	6.38%
Monthly	0%	NA	2.13%
Unknown	0%	NA	0%
Use of air conditioning	86.54%	66.04%	87.23%
Music during work	71.15%	50.94%	82.98%
Exposure loud noises *			
Motors, planes, boats	5.77%	1.89%	8.52%
Loud music	7.69%	15.09%	27.66%
Firearms	1.92%	1.89%	0%
Gardening tools	15.38%	15.09%	2.13%
Fireworks/explosions	0%	0%	2.13%
Others	5.76%	5.66%	0%
No exposure	67.31%	67.92%	68.09%
Ototoxic agents	0%	5.66%	0%
**Part 4. Annoyance**			
Annoyance (mean ± SD)	2.79 ± 0.91	2.06 ± 0.84	2.91 ± 0.9
Increasing annoy. with age	40.38%	7.55%	12.77%
Profession alters hearing	32.69%	0%	8.51%
Patients fearing noise	59.62%	NA	38.3%
Complaints profession *			
No complaints	36.54%	67.92%	34.04%
Tinnitus	7.69%	1.89%	6.38%
Intolerance to noise	28.85%	1.89%	14.89%
Speech in quiet	0%	1.89%	4.26%
Speech in adverse conditions	32.69%	32.08%	21.28%
Stress	9.62%	5.66%	8.51%
Fatigue	15.38%	5.66%	4.26%
Headache	5.77%	3.77%	27.66%
Nervous/irritation	15.38%	9.43%	14.89%
Others	1.92%	0%	0%
**Part 5. Knowledge and interest**			
Knowledge HL (mean ± SD)	1.9 ± 0.82	1.77 ± 0.93	2.13 ± 0.9
Information HL	96.15%	90.57%	97.87%
**Part 6. Preventive measures**			
Hearing protection? *			
Disposable earplugs	1.92%	11.32%	4.26%
Silicon plugs	0%	3.77%	2.13%
Wax plugs	0%	1.89%	0%
Ear muffs	0%	5.66%	0%
Universal earbuds with filter	1.92%	1.89%	2.13%
Otoplastics	5.77%	3.77%	17.02%
No hearing protection	90.38%	75.47%	78.72%
Use of hearing protection? *			
At work	1.92%	0%	14.89%
In private	7.69%	24.53%	6.38%
Sound absorbing material	21.15%	3.77%	10.64%
Extractor systems separated	92.31%	NA	51.06%
Hearing tested?	5.77%	13.21%	0%

* Multiple answers possible. HL—hearing loss.
